# Immune response of rats vaccinated orally with various plant-expressed recombinant cysteine proteinase constructs when challenged with *Fasciola hepatica* metacercariae

**DOI:** 10.1371/journal.pntd.0005451

**Published:** 2017-03-23

**Authors:** Malgorzata Kesik-Brodacka, Agnieszka Lipiec, Monika Kozak Ljunggren, Luiza Jedlina, Katarzyna Miedzinska, Magdalena Mikolajczak, Andrzej Plucienniczak, Andrzej B. Legocki, Halina Wedrychowicz

**Affiliations:** 1 Department of Bioengineering, Institute of Biotechnology and Antibiotics, Warsaw, Poland; 2 Division of Parasitology, Faculty of Veterinary Medicine, Warsaw University of Life Sciences, Warsaw, Poland; 3 Witold Stefański Institute of Parasitology, Polish Academy of Sciences, Warsaw, Poland; 4 Institute of Bioorganic Chemistry, Polish Academy of Sciences, Poznan, Poland; Queen's University Belfast, UNITED KINGDOM

## Abstract

**Background:**

Cysteine proteinases of *Fasciola hepatica* are important candidates for vaccine antigens because of their role in fluke biology and host-parasite relationships. In our previous experiments, we found that a recombinant cysteine proteinase cloned from adult *F*. *hepatica* (CPFhW) can protect rats against liver fluke infections when it is administered intramuscularly or intranasally in the form of cDNA. We also observed considerable protection upon challenge following mucosal vaccination with inclusion bodies containing recombinant CPFhW produced in *Escherichia coli*.

In this study, we explore oral vaccination, which may be the desired method of delivery and is potentially capable of preventing infections at the site of helminth entry. To provide antigen encapsulation and to protect the vaccine antigen from degradation in the intestinal tract, transgenic plant-based systems are used.

**Methodology:**

In the present study, we aimed to evaluate the protective ability of mucosal vaccinations of 12-week-old rats with CPFhW produced in a transgenic-plant-based system. To avoid inducing tolerance and to maximise the immune response induced by oral immunisation, we used the hepatitis B virus (HBV) core protein (HBcAg) as a carrier. Animals were immunised with two doses of the antigen and challenged with 25 or 30 metacercariae of *F*. *hepatica*.

**Conclusions:**

We obtained substantial protection after oral administration of the plant-produced hybrids of CPFhW and HBcAg. The highest level of protection (65.4%) was observed in animals immunised with transgenic plants expressing the mature CPFhW enzyme flanked by Gly-rich linkers and inserted into c/e1 epitope of truncated HBcAg. The immunised rats showed clear IgG1 and IgM responses to CPFhW for 4 consecutive weeks after the challenge.

## Author summary

Infection with *Fasciola hepatica*, a liver fluke, is one of the most significant veterinary problems due to the worldwide distribution of this parasite, a wide spectrum of host organisms and the resulting economic loss. Human fasciolosis caused by *F*. *hepatica* is recognised by the World Health Organization as an important emerging but neglected tropical disease. Development of an effective vaccine against this disease is becoming a priority, especially as the appearance of drug-resistant strains undermine the currently employed drug-based treatments. The two primary issues when developing a vaccine are the selection of an appropriate vaccine antigen and the route of antigen administration. In our studies, we use one of the *F*. *hepatica* cysteine proteinases, which are promising antigens for vaccine construction. We evaluate the immunogenicity and protective ability of various modifications of this cysteine proteinase produced in plants. We show that substantial protection can be obtained when plant-expressed hybrid proteins are administered orally.

## Introduction

Infection with *Fasciola hepatica*, a liver fluke, is one of the most significant veterinary problems due to the worldwide distribution of this parasite and a wide spectrum of host organisms [1]. Fasciolosis causes economic losses of US$3 billion annually due to its impact on livestock production, thereby affecting the food industry worldwide [2,3]. In recent years, the number of *F*. *hepatica* infections has dramatically risen, a trend that has been attributed to climate change [4,5]. The prevalence of fasciolosis has increased by up to 12-fold in the EU member states during recent years [6]. Human fasciolosis caused by *F*. *hepatica* is recognised by WHO as an important emerging but neglected tropical disease, with estimates of 2.4–17 million people infected worldwide, and approximately 180 million living at risk of infection [7,8]. Large endemic areas have been described in Peru [9,10], Egypt [11], Iran [12,13], North America [14], Pakistan [15] and other regions, with prevalences of 72–100% in the Bolivian Altiplano [16].

Currently, the treatment of fasciolosis is based primarily on the use of chemotherapy [17]. As *F*. *hepatica* drug resistance becomes more frequent, it is possible that there will be more cases of infection in humans with drug-resistant *F*. *hepatica*, which poses a real problem for the treatment of human fasciolosis [18]. The emergence of drug-resistant parasites [19–23], combined with the growing consumer concern over chemical residues in food and their passage into the environment, have prompted the need for novel means of disease control [24]. The most effective method of parasite control is vaccination [3,25]. It is strongly believed that the control of the human infection would greatly benefit from vaccines targeting the animal infection [26]. A number of experimental, parenterally administered vaccines against fasciolosis have shown that the development of a successful commercial vaccine still remains a challenge [26–29].

There are two primary issues that need to be addressed when developing a vaccine. The first is the selection of an appropriate vaccine antigen. Several immunogenic fluke antigens have been identified; among them, the most promising appears to be a cysteine proteinase (cathepsin L) [27,30–32]. We previously showed that the cDNA of this *F*. *hepatica* cysteine proteinase, administered intramuscularly or intranasally, induces a protective immune response when delivered prior to the infection with fluke metacercariae (mc) [33,34]. We also observed considerable protection and reduced pathology upon challenge following parenteral and mucosal vaccination with inclusion bodies containing recombinant cysteine proteinase cloned from adult *F*. *hepatica* (CPFhW) and produced in *Escherichia coli* [35,36].

Another important aspect of vaccine development is the route of antigen administration. As the intestinal tract is the location where invasion of the fluke begins, it is postulated that the host protective response should occur in the intestinal mucosa-associated lymphoid tissue. It has previously been shown that challenge infections in immunised rats are rejected at the level of the gut and peritoneum in the first few days after infection [37–39]. Once the fluke reaches the liver and bile ducts, it seems to be impervious to protective immune mechanisms [40]. Therefore, oral vaccination is the desired route of delivery to prevent infections at the site of pathogen entry. Unfortunately, vaccines administered into the intestinal tract may be ineffective due to the rapid degradation of the vaccine antigens by intestinal proteases. It has been speculated that the plant cell wall delays the digestion of plant-produced and delivered antigens [41].

Therefore, oral vaccination is the desired route of antigen delivery, and plant-based delivery vehicles may increase the amount of antigen presented to the gut-associated lymphoid tissue [42]. Additionally, production of antigen in plants for oral vaccination eliminates the need for purification, cold storage, transportation and sterile delivery. In our previous study [43], we reported that feeding mice with lettuce expressing the cysteine proteinase from *F*. *hepatica* is effective in inducing a specific antibody response against this antigen.

The aim of the present study was to evaluate the immunogenicity and protective ability of various modifications of plant-produced *F*. *hepatica* cysteine proteinase in oral vaccination. As a carrier for the *F*. *hepatica* antigen, we used hepatitis B virus (HBV) core protein (HBcAg). The carrier was applied to avoid tolerance and to potentiate the immune response induced by oral immunisation. We showed that substantial protection can be obtained when the plant-expressed hybrid proteins are orally administered.

## Materials and methods

### Ethics statement

All experimental protocols were approved by the III Local Animal Experimentation Ethics Committee of Warsaw University of Life Sciences, approval number: 39/2003. The experiments were performed according to the guidelines of European Communities Council Directive (86/609/EEC). All efforts were made to minimise animal suffering and to reduce the number of animals used.

### Vaccination antigens

#### Vectors for protein expression in plants

The pcDNA3.1 plasmid carrying cDNA encoding the *F*. *hepatica* cysteine proteinase [33,34] provided the coding sequence of CPFhW. The plasmid comprising the entire HBV (subtype ayw4) genome was used as the source of the region encoding truncated (1 to 149 aa) HBV core protein (HBcAg(T)). *Saccharomyces cerevisiae* DNA was used as the source of the ubiquitin sequence. The nucleotide numbering for the CPFhW, HBcAg(T), and ubiquitin sequences refer to sequences deposited in GenBank, accession no. AY277628, Z35716, and X05731, respectively.

**Fig 1:**
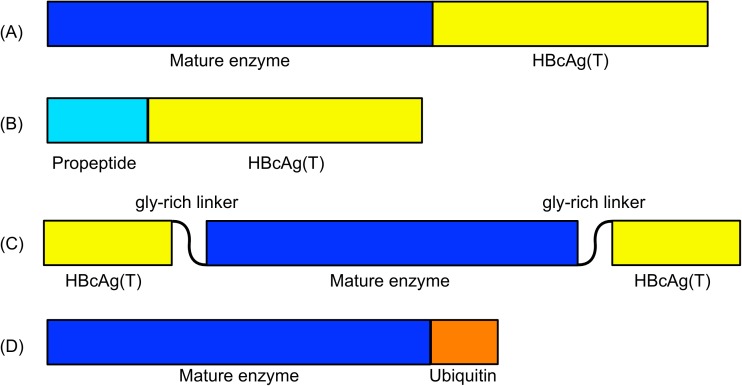
Schematic representation of the construct encoding fusion proteins. (A) mCPFhW::C construct consists of the sequence encoding mature CPFhW (nt 319–976) ligated to the 5' end of the sequence encoding HBcAg(T) (nt 1903–2451). (B) pCPFhW::C construct consists of the sequence encoding the propeptide of CPFhW (nt 46–318) ligated to the 5' end of the sequence encoding HBcAg(T). (C) mCPFhW::G::C construct consists of the sequence encoding HBcAg(T) with an insertion encoding the mature CPFhW flanked by Gly-rich linkers ((Gly)_4_-Ser-(Gly)_4_-Gln-(Gly)_2_). The mature CPFhW flanked at both ends by glycine residues is ligated between nt 2120 and 2151 of the sequence encoding HBcAg(T). (D) U::mCPFhW construct consists of a ubiquitin sequence spanning nt 767–995 ligated to the 5' end of the sequence encoding mature CPFhW.

Four constructs encoding the fusion proteins were obtained: mCPFhW::C, pCPFhW::C, mCPFhW::G::C, and U::mCPFhW (Fig 1A–1D).

The obtained hybrid constructs were placed under the transcriptional control of the 35S constitutive promoter of cauliflower mosaic virus in the pROK2 plant expression vector. Standard protocols were used for PCR, restriction digests, ligations, and transformations. The validity of the sequences of hybrid genes introduced into the pROK2 plasmid was confirmed by sequencing. The recombinant vectors were introduced into the strain LBA 4404 of *Agrobacterium tumefaciens*, which was used to transform lettuce (*Lactuca sativa*).

Leaves from the transgenic plants were lyophilised before being fed to the experimental animals. The amount of vaccine antigen in the lyophilised plant tissue was calculated based on quantitative enzyme-linked immunosorbent assay (ELISA) results [43]. Antibodies used in ELISAs were produced against the same antigens as the ones we used in this experiment. Antigens for antibody production were expressed in *E*. *coli*. The amount of antigen content was measured for each batch of lyophilised transgenic plants.

### Parasites

*F*. *hepatica* mc were obtained from experimentally infected intermediate hosts, a laboratory strain of *Galba truncatula* snails reared at W. Stefański Institute of Parasitology. Miracidia were cultured from the *F*. *hepatica* eggs obtained from the gall bladders of naturally infected cattle slaughtered in the slaughterhouse. Liver and gall bladders were classified as waste because of the presence of pathological changes caused by the fluke invasion. There was no need to apply for permission to use them. Each snail was exposed to two recently hatched miracidia for approximately 24 h at room temperature. After exposure, the snails were maintained in Petri dishes and fed *Oscillatoria* algae. Mc were collected starting from day 70 after the exposure of the snails to miracidia. To collect the mc, the infected snails were placed in Petri dishes lined with transparent cellophane and exposed to light for up to 2 h. The mc were stored in water at 4°C for at least 2 weeks before use.

### Experimental animals

Inbred 12-week-old Sprague-Dawley (SPRD/Mol/Lod) male rats were used in the experiments. The animals were housed in groups and randomly assigned to the treatment or control groups. The rats were acclimated for 1 week before the experiments were initiated. They were provided with food and water ad libitum until 16 h before being fed the antigen and challenged with mc, during which period they were deprived of food [39].

### Vaccination procedure

Three separate experiments were carried out using various CPFhW-based antigens expressed in plants (Table 1). In all these experiments, two doses of transgenic lettuce were intragastrically administered to the vaccinated rats in 4-week intervals. For each vaccination, 1 g of lyophilised lettuce was used, which corresponds to 10 μg of the antigen [43]. In each experiment, the group of control rats was mock-immunised with the lyophilised control lettuce according to the same schedule as that used for the experimental animals. Twenty-eight days after the second antigen dose, each rat was orally challenged with mc of *F*. *hepatica* in 1 ml of water.

**Table 1:** Experimental design.

Exp. No.	Group	No. of rats	Antigen	Challenge
I	1	8	2 x 1 g of lyophilised lettuce expressing the mCPFhW::C	25 mc
I	2	8	2 x 1 g of lyophilised lettuce expressing the pCPFhW::C	25 mc
I	3	8	2 x 1 g of lyophilised control lettuce	25 mc
II	1	8	2 x 1 g of lyophilised lettuce expressing the mCPFhW::G::C	30 mc
II	2	8	2 x 1 g of lyophilised control lettuce	30 mc
III	G	12	2 x 1 g of lyophilised lettuce expressing the mCPFhW::G::C	30 mc
III	U	12	2 x 1 g of lyophilised lettuce expressing the U::mCPFhW	30 mc
III	C	12	2 x 1 g of lyophilised control lettuce	30 mc
III	N	12	none	none

After administration, the syringe and cannula were flushed with 0.5 ml of water to recover any remaining mc. In Experiment I, 25 mc were used. In Experiments II and III, the viability of the metacercariae was lower. Therefore, in order to maintain the experimental conditions, the number of metacercariae was increased proportionally to the number of that observed for in vitro excystment of this *F*. *hepatica* isolate.

In Experiments I and II, all rats were euthanised and dissected five weeks after the challenge infection. In Experiment III, four rats from each group were euthanised and dissected on days 7, 35 and 63 after the challenge.

Livers were removed for the evaluation of macroscopic lesions namely the hepatic fibrosis caused by the migration of *F*. *hepatica* through the liver parenchyma. Hepatic damage due to the invading parasite was evaluated subjectively by observing macroscopic alterations in the organ based on a number of criteria, including the following: colour change to greyish-white, increase in size, change in consistency, dilatation and thickening of bile ducts, and formation of surface scars [44]. The degree of lesions observed (index of liver damage) was summarised semiquantitatively using the following scale to express the intensity and extent of the alteration (tissue necrosis or liver nodules) observed: “0”, no visible sign of tissue necrosis or liver nodules; “1”, mild liver necrosis; “2”, moderately mild liver damage of up to 15% of the liver surface; “3”, moderate liver damage—approximately 30% of the liver surface; “4”, intense liver damage of up to 50% of the liver surface; “5”, severe liver necrosis with >50% of the liver surface showing pathological changes. Index of liver damage and the intensity of the fluke invasion (no. of flukes found during the dissection) was estimated on days 35 and 63 after the challenge.

To recover flukes from the parenchyma and biliary tree, the livers were stored at 37°C in separate Petri dishes containing RPMI-1640 cell culture medium. Blood, peritoneal fluid samples, and mesenteric lymph nodes were also collected in Experiment III to compare the cellular and antibody responses of vaccinated and control rats during the challenge infection.

### Flow cytometry analysis

The peritoneal and mesenteric lymph nodes from each rat were collected aseptically. Each tissue was immersed in RPMI-1640 cell culture medium (4°C) supplemented with 2% heat-inactivated foetal bovine serum. The tissues were cut into 1 mm^3^ pieces with sterile scalpels. The tissue fragments were placed in a Petri dish containing sterile wash medium on ice. The suspended cells were then washed and quantified using a haemocytometer. Cell viability was determined by trypan blue exclusion. The counted cells were centrifuged for 5 min at 1200 rpm and 4°C, followed by resuspension in PBS containing 0.05% sodium azide and incubation on ice for 15–30 min. The cells were pelleted by centrifugation and resuspended. For these and peripheral blood samples, the quantities of eosinophils and monocytes and the phenotype of the T cells (CD4+ and CD8+) were investigated by using a panel of monoclonal anti-rat antibodies (BD Pharmingen). Monoclonal antibodies against rat CD4 (clone: OX-38) receptors were labelled with phycoerythrin, and the CD8 antibodies (clone: OX-8) were labelled with fluorescein isothiocyanate. The cells incubated with corresponding isotype control (BD Pharmingen) mouse IgG2a, κ labelled with phycoerythrin (clone: G155-178) and mouse IgG1, κ labelled with fluorescein isothiocyanate (clone: MOPC-31C) antibodies were used as controls for nonspecific antibody binding to the cells. For FACS analysis, single-cell suspensions (50 μl) were incubated with mAbs and washed. Subsequently, red blood cells were lysed in FACS Lysing Buffer (Becton Dickinson), and leukocytes were analysed using a FACSCalibur flow cytometer (Becton Dickinson) with an argon excitation source. Data acquisition was performed using CellQuest software (Becton Dickinson). The results were expressed as the percentage of total mononuclear cells from a designated region. Leukocytes were identified by their characteristic appearance on a dot plot of FSC versus SSC and electronically gated to exclude platelets and dead-cell debris. Eosinophils are autofluorescent, and this property was used to identify them. Lymphocytes were selected using fluorescence-labelled antibody specific for the antigen as well as their phenotypic and morphometric features.

### ELISA test

Microtiter plates (MaxiSorb) were coated with fluke CPFhW [35] or ES [34] antigen (15 μg/ml of 0.05 M carbonate-bicarbonate buffer at pH 9.6). The plates were incubated overnight at 4°C and subsequently washed four times with 10 mM Tris/0.15 M NaCl at pH 7.4 (TBS) containing 0.05% Tween 40. The excess binding sites were blocked by washing with 100 μl/well of TBS and 4% skimmed milk for 2 h at room temperature. The serum samples (diluted 1:100) were added to each well, and the plates were incubated for 30 min at 37°C. Each serum sample was tested in triplicate. Following three washes, HRP-conjugated anti-rat IgG1, IgA, and IgM monospecific antisera (Bio-Rad formerly AbD Serotec) were added to each well, and the plates were incubated for 30 min at 37°C. The plates were washed three times, and the binding of the conjugates was visualised with 3,3,5’,5’-tetramethylbenzidine in 0.1 M sodium citrate, pH 4.5, containing 0.03% H_2_O_2_. The reaction was stopped, and the absorbance was measured at 405 nm using an MRX ELISA Reader (Dynatech Laboratories). For the detection of the IgE antibodies, the sera were diluted 10-fold, and a monoclonal mouse antibody specific to rat IgE (ICN Immunobiologicals) was used [45]. Peroxidase-labelled anti-mouse Ig (ICN Immunobiologicals) was used as the secondary antibody.

### Statistical analysis

The data are expressed as the mean ± the standard deviation for each experimental group. One-way ANOVA with parametric F-test was used to compare the results of the cell count and antibody levels between groups after the challenge in Experiment III. Mann-Whitney *U*-tests were used to compare the number of flukes recovered from vaccinated and challenge control rats. *p*-values < 0.05 were considered statistically significant.

The percent protection for vaccinated animals was calculated as (1 − *V*/*C*) × 100, where C is the mean burden of the control animals challenged with mc, and V is the mean burden of the immunised rats challenged with mc [46].

### Accession numbers

Genetic sequences used in this study are deposited in GenBank with accession numbers: CPFhW, AY277628; HBcAg, Z35716; Ubiquitin, X05731.

## Results

In the conducted experiments, we evaluated the protective ability and immunogenicity of various modifications of plant-produced *F*. *hepatica* cysteine proteinase by oral vaccination. To be effective as vaccines, monomeric proteins usually require chemical coupling to high molecular weight carriers or they need to be applied together with adjuvants [47]. In our approach, hepatitis B virus core protein (HBcAg) was used to avoid tolerance and to enhance the immune response. This subviral particle is highly immunogenic in humans and experimental animal models. We verified the effectiveness of HBcAg to potentiate humoural and cellular immune responses against CPFhW fused to this protein. For this purpose, several DNA expression vectors were prepared. The constructs encode hybrids of *F*. *hepatica* cysteine proteinase (CPFhW) and the truncated HBV core protein (HBcAg (T)), thereby enabling the expression of the mature CPFhW enzyme fused to HBcAg(T), the propeptide of the CPFhW enzyme fused to HBcAg(T), the HBcAg(T) with an insertion encoding the mature CPFhW enzyme flanked by Gly-rich linkers ((Gly)_4_-Ser-(Gly)_4_-Gln-(Gly)_2_), and ubiquitin fused to mature CPFhW (Fig 1A–1D). To obtain the transgenic plants, the vectors were introduced into the strain LBA 4404 of *Agrobacterium tumefaciens*, which was used to transform lettuce (*Lactuca sativa*). Lettuce is characterised by fast growth and a good crop yield. These characteristics allow the procurement of large amounts of genetic material within approximately 3 months, i.e., a relatively short time for a plant-based system. The expression levels of the hybrid proteins varied between individual plants. The ELISA test revealed that the expression ranged from undetectable to approximately 20 μg/g wet weight. The highest expression levels were obtained for the antigen fused to ubiquitin. Such high accumulation was most likely due to the fusion with the ubiquitin gene.

For each vaccination, 1 g of lyophilised lettuce was used, which relates to 10 μg of the antigen [43]. For this purpose, only the plants expressing each of the antigens so that 1 g of lyophilisate contains 10 μg of the antigen were selected for immunisations. The lyophilised and powdered leaves of plants expressing the vaccination antigens were fed to the experimental animals.

### Fluke burden

Three experiments were conducted to compare the efficacy of oral administration of lyophilised lettuce containing the antigen variants. The worm burdens and indexes of liver damage following the challenge are shown in Table 2.

**Table 2:** Results of the experiments.

Exp. No.	Group	Antigen	No. of flukes found during dissection (mean+/-SE)	Protection (%)	Index of liver damage
I	1	lettuce expressing mCPFhW::C	2,1,3,2,2,1,2,1(1.75+/-0.25)	64	2
I	2	lettuce expressing pCPFhW::C	3,2,3,1,3,4,3,1(2.5+/-0.38)	49	3
I	3	control lettuce	5,4,6,5,5,6,3,54.88+/-0,29	0	4
II	1	lettuce expressing mCPFhW::G::C	1,3,1,2,2,2,3,4(2.25+/-0.36)	65.4	2
II	2	control lettuce	8,6,5,7,8,4,8,6(6.5+/-0.53)	0	5
III	G	lettuce expressing mCPFhW::G::C	1,2,3,5,0,0,4,3(2.25+/-0.65)	62.5	1
III	U	lettuce expressing U::mCPFhW	2,7,3,1,4,5,0,2(3+/-0.8)	50	3
III	C	control lettuce	4,9,8,5,4,6,4,8(6+/-0.73)	0	5
III	N	none			

Statistical evaluation of differences in the fluke burden among groups of vaccinated rats and vaccinated and challenge control rats (Table 2)

Exp. I:

Group 1 vs. Group 3: p<0.0001

Group 1 vs. Group 2: p<0.001

Group 2 vs. Group 3: p<0.0001

Exp. II

Group 1 vs. Group 2: p<0.0001

Exp. III

Group G vs. C: p<0.001

Group U vs. G: p<0.1

Group U vs. C: p<0.001

In all conducted experiments (I, II, III), a profound reduction in the fluke burden was found in the vaccinated rats. In addition, the livers of the vaccinated rats were not as damaged as those of the challenge controls. Among the used antigens, the mature CPFhW enzyme fused to HBcAg(T) was found to provide the highest degree of protection.

In Experiment I, the highest degree of protection (64%) was observed in rats orally fed lyophilised lettuce expressing the sequence encoding the mature CPFhW enzyme fused to HBcAg(T) (Group 1). Rats immunised with lettuce expressing the propeptide of CPFhW enzyme (Group 2) showed significantly (p<0.05) lower protection than observed in the group immunised with the mature CPFhW (Group 1).

In Experiment II, 65.4% of protection was observed in the rats vaccinated with lettuce expressing HBcAg(T) with an insertion encoding the mature CPFhW enzyme flanked by Gly-rich linkers (mCPFhW::G::C).

In the next Experiment (Experiment III), where a new batch of plants expressing the same antigen (mCPFhW::G::C) was used, the level of protection was slightly lower (62.5%) than in Experiment II.

Lower protection was observed when the rats were immunised with lettuce transformed with mature CPFhW enzyme not fused to HBcAg(T) (Group U). When the rats were immunised with lettuce transformed with the U::mCPFhW construct (Experiment III, Group U), the observed protection level was lower than when the construct containing mature CPFhW fused with HBcAg(T) was used (Experiment I Group 1, Experiment II Group 1 and Experiment III Group G).

### Serum antibody responses of vaccinated and control rats to challenge infection

The humoural response to *F*. *hepatica* antigens was examined by comparing the antibody OD values of the control and vaccinated groups using one-way ANOVA.

**Fig 2:**
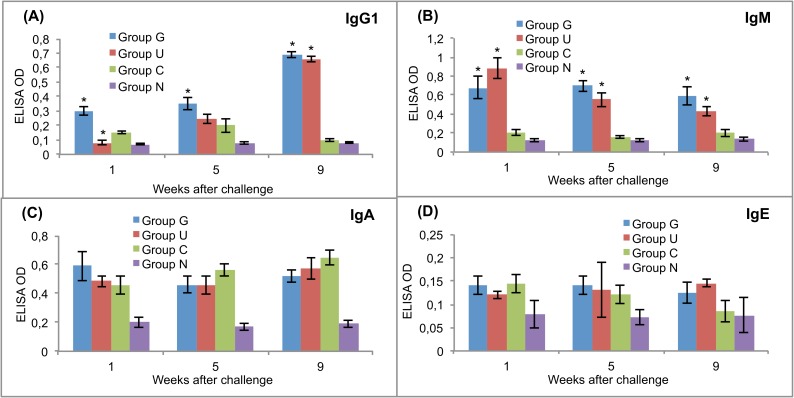
Post-challenge serum antibody isotype responses of vaccinated and control rats to recombinant cysteine proteinase. (A) IgG1. (B) IgM. (C) IgA. (D) IgE. Group G–Lyophilised lettuce expressing the mature CPFhW enzyme flanked by Gly-rich linkers; Group U–Lyophilised lettuce expressing the mature CPFhW protein fused with ubiquitin; Group C–Lyophilised control lettuce; Group N–None. At each timepoint, four rats from each group were euthanised and dissected. *Indicates significantly increased numbers of cells (*p*<0.05). Error bars indicate standard deviation.

**Fig 3:**
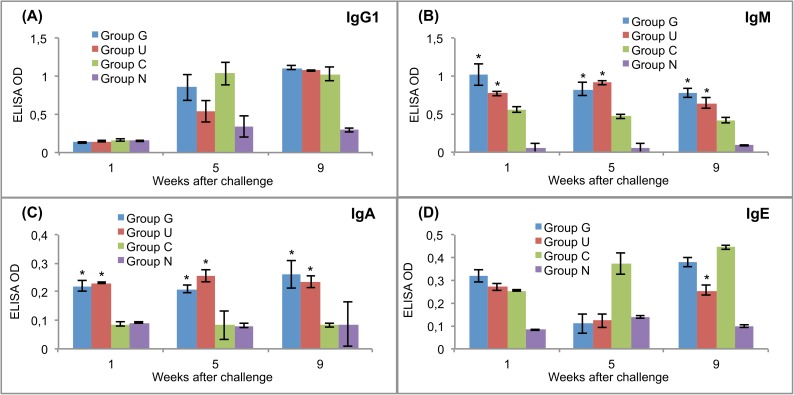
Post-challenge serum antibody isotype responses of vaccinated and control rats to ES products of adult flukes. (A) IgG1. (B) IgM. (C) IgA. (D) IgE. Group G–Lyophilised lettuce expressing the mature CPFhW enzyme flanked by Gly-rich linkers; Group U–Lyophilised lettuce expressing the mature CPFhW protein fused with ubiquitin; Group C–Lyophilised control lettuce; Group N–None. At each timepoint, four rats from each group were euthanised and dissected. *Indicates significantly increased numbers of cells (*p*<0.05). Error bars indicate standard deviation.

The levels of antibodies against CPFhW and ES antigens were measured in the sera of experimental rats after the challenge infection. All serum samples were tested in triplicate. The results are presented in Figs 2 and 3.

Rats in group G showed a significantly higher IgG1 response than the rats in groups U and C at weeks 1 and 5 after the challenge infection (Fig 2A). After the challenge, the CPFhW-specific IgM antibodies in both vaccinated groups showed significantly (*p*<0.05) higher OD values than the antibodies in the challenge controls (Fig 2B). No statistically significant difference between the vaccinated and challenge control groups were observed with respect to the CPFhW-specific IgA and IgE levels (Fig 2C and 2D, respectively). IgM and IgA targeting ES antigens were present at significantly (*p*<0.05) higher levels in the vaccinated rats than in the challenge controls (Fig 3B and 3C).

### Cellular response in blood, peritoneal cavity and mesenteric lymph nodes

**Fig 4:**
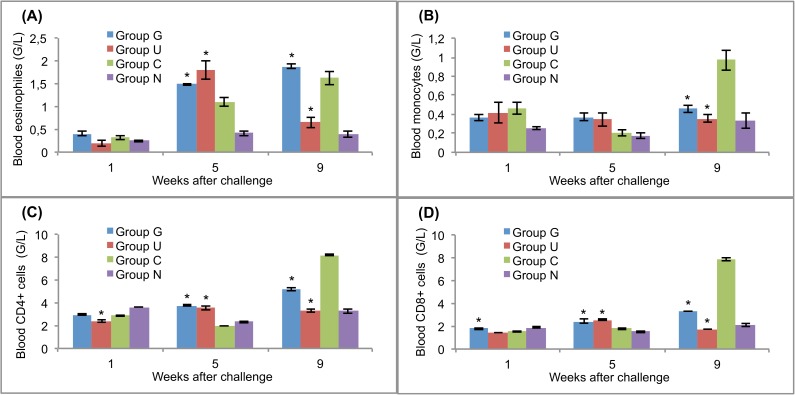
Leukocyte responses in the blood of vaccinated and control rats to challenge infections. (A) eosinophils. (B) monocytes. (C) CD4+ T lymphocytes. (D) CD8+ T lymphocytes. Group G–Lyophilised lettuce expressing the mature CPFhW enzyme flanked by Gly-rich linkers; Group U–Lyophilised lettuce expressing the mature CPFhW protein fused with ubiquitin; Group C–Lyophilised control lettuce; Group N–None. At each timepoint, four rats from each group were euthanised and dissected. *Indicates significantly increased numbers of cells (*p*<0.05). Error bars indicate standard deviation.

**Fig 5:**
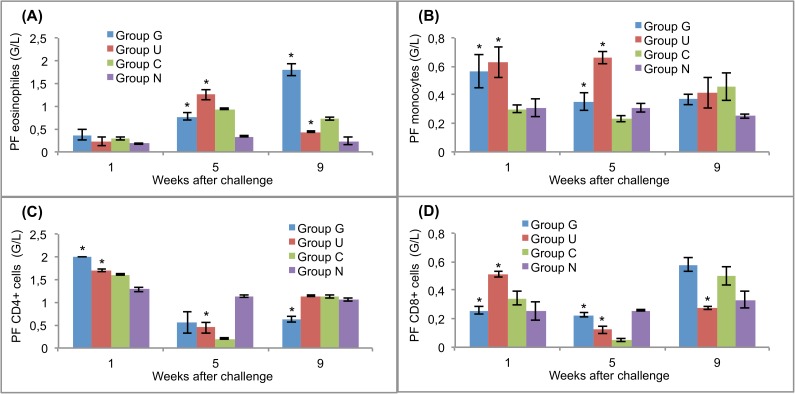
Leukocyte responses in the peritoneal cavity of vaccinated and control rats to challenge infections. (A) eosinophils. (B) monocytes. (C) CD4+ T lymphocytes. (D) CD8+ T lymphocytes. Group G–Lyophilised lettuce expressing the mature CPFhW enzyme flanked by Gly-rich linkers; Group U–Lyophilised lettuce expressing the mature CPFhW protein fused with ubiquitin; Group C–Lyophilised control lettuce; Group N–None. At each timepoint, four rats from each group were euthanised and dissected. *Indicates significantly increased numbers of cells (*p*<0.05). Error bars indicate standard deviation.

**Fig 6:**
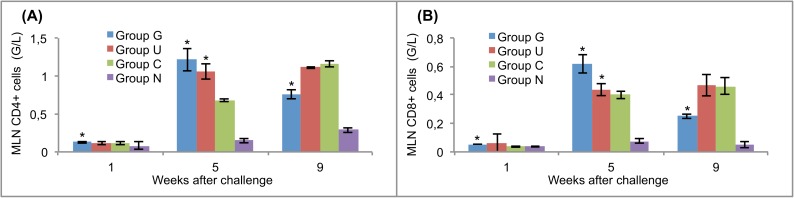
T lymphocyte responses in the mesenteric lymph nodes of vaccinated and control rats to challenge infections. (A) CD4+ cells. (B) CD8+ cells. Group G–Lyophilised lettuce expressing the mature CPFhW enzyme flanked by Gly-rich linkers; Group U–Lyophilised lettuce expressing the mature CPFhW protein fused with ubiquitin; Group C–Lyophilised control lettuce; Group N–None. At each timepoint four rats from each group were euthanised and dissected. *Indicates significantly increased numbers of cells (*p*<0.05). Error bars indicate standard deviation.

The challenge with *F*. *hepatica* mc caused remarkable changes in the numbers of eosinophils, monocytes, CD4+ cells and CD8+ cells in both the vaccinated and challenge control rats (Figs 4–6). Significantly (*p*<0.05) increased numbers of blood eosinophils were observed in the vaccinated rats and challenge controls at 5 weeks after challenge and in groups G and C at 9 weeks after challenge (Fig 4A). Similar cellular response dynamics were observed for the peritoneal fluid (Fig 5A). Blood monocytes were present at similar levels in the vaccinated and control rats at weeks 1 and 5 after challenge, but at the end of the experiment, the rats in group C had the highest numbers of these cells (Fig 4B). In the peritoneal fluid, the numbers of these cells were significantly (*p*<0.05) higher than that in the challenge controls at the first and fifth weeks after the challenge infection (Fig 5B).

CD4+ and CD8+ lymphocytes appeared in the blood of the vaccinated rats in significantly (*p*<0.05) higher numbers than in controls on day 35 after infection. In contrast, at week 9, the highest level of these cells was found in the animals of group C (Fig 4C and 4D). In the peritoneal fluid, the highest number of CD4+ cells was found in rats in group G on day 7 post-infection; however, the number of CD8+ cells at the same time was significantly (*p*<0.05) lower than in group U (Fig 5C and 5D). At the end of the experiment, the opposite situation was observed. The peritoneal fluid from both vaccinated and challenge control rats contained significantly fewer CD4+ and CD8+ cells than the peritoneal fluid from uninfected control rats on day 35 post-infection (Fig 5C and 5D). Mesenteric lymph nodes showed no CD4+ response on day 7 after the challenge (Fig 6A), whereas the CD8+ cells reached the highest level in group U at that time (Fig 6B).

## Discussion

Since the early 1990s, edible vaccines based on transgenic plants have been investigated. Transgenic plants offer several features that make them an attractive platform for the delivery of oral vaccine antigens [48]. When an antigen is produced in edible plants or parts thereof, the production cost of a vaccine can be reduced considerably, thus increasing its availability and usability for both humans and animals [42]. Furthermore, the production of vaccines in plants eliminates the risk of contamination with animal pathogens such as viruses and prion proteins [49].

Transgenic plants serve as bioreactors for antigen production and provide a natural built-in system for antigen encapsulation [50]. The data from the literature suggest that when administered orally, the antigen immunogenicity and biological activities are preserved in the harsh environment of the gastrointestinal tract due to their natural bioencapsulation in the plant cell. It has been demonstrated that bioencapsulation influences the prolonged release and presentation of the antigen to immune responsive sites [51]. Once these vaccines pass through the gastric environment and reach the small intestine, they are taken up by the M cells for the induction of mucosal and systemic immune responses [52,53].

The results presented in this study clearly indicate that substantial protection can be obtained when plant-produced heterologous proteins (hybrids of CPFhW and HBcAg(T)) are administered with food. The administration of durable powdered lyophilised plant tissue made possible to concentrate and standardise the plant-bioencapsulated antigen content in the vaccine doses. The HBV core protein (HBcAg(T)) was used as a carrier to enhance the immune response. This protein forms symmetrical structures, contains potent T helper epitopes and is able to activate B cell production of anti-HBcAg immunoglobulins both in the presence and absence of antigen-specific T cells. Therefore, the HBcAg(T) has gained interest as a carrier system to potentiate humoural and cellular immune responses against heterologous epitopes fused to this protein [47,54].

Foreign heterologous epitopes can be inserted at different positions within the HBcAg protein. It has been shown that it is possible to fuse HBcAg with large inserts without disrupting its ability to confer immunogenicity [55]. Ravin et al. [56] used HBcAg as a carrier for the ectodomain of influenza virus matrix protein 2 (M2e). In their experiment, the M2e peptide was fused to the N-terminus of the HBcAg protein, and the chimeric protein was expressed in plants. This plant-produced antigen was highly immunogenic in mice, and the mice were protected against lethal influenza challenges. Fusion to both the N-terminus and the C-terminus is compatible with the assembly of the HBcAg particle and preserves its native antigenicity and immunogenicity. However, fusion to an immunodominant internal site of HBcAg (c/e1 epitope) reduces its antigenicity and immunogenicity, while dramatically enhancing the immunogenicity of the inserted foreign epitope [57]. In the construct mCPFhW::G::C, the DNA sequence of mature *F*. *hepatica* CPFhW enzyme was inserted into the c/e1 epitope of HBcAg(T), which is located around amino acid 80 [58]. The original core protein amino acids were removed, including Asp-78, Pro-79 and Ala 80. To minimise steric constraints, Gly-rich linkers were added between the mature CPFhW enzyme and the remaining regions of HBcAg(T). Gly-rich linkers were used because glycine has low preference to form an α-helix, and its lack of sidechain maximises the freedom of the polypeptide backbone conformation [59]. We observed the highest level of protection (65.4%) after the administration of transgenic plants expressing this hybrid protein; however, in rats vaccinated with hybrid protein (mCPFhW::HBcAg(T)) lacking the Gly-rich linkers, the protection level was only marginally lower (64%).

The mature cysteine proteinase is an enzyme that is secreted by *F*. *hepatica* and with which the host organism has contact. This contact can explain why immunisation with this antigen resulted in the best protection in the challenged animals, especially in comparison to immunisation with the propeptide form of the CPFhW protein. Although the propeptides of cathepsin L-like proteins have been predicted to contain important B cell epitopes [60], immunisation with this antigen variant resulted in lower protection values than immunisation with the mature protein.

Obtaining a high expression level of recombinant proteins in transgenic plants is a well-known challenge. Therefore, attaining a high expression level of the recombinant protein is one of the primary design objectives when preparing DNA constructs. The data from the literature suggest that the presence of the ubiquitin promoter or ubiquitin gene can significantly augment the expression of the gene fused to ubiquitin [61]. The yield enhancement has been proposed to be due to a chaperone effect exerted by the ubiquitin. In our experiments, we used the U::mCPFhW construct, consisting of the ubiquitin sequence ligated to the 5' end of the sequence encoding the mature CPFhW, to potentiate the expression of the mature CPFhW. As a result, we obtained the expression of mature CPFhW as a translational fusion with ubiquitin. In our study, the expression of the mature CPFhW fused to ubiquitin allowed for the accumulation of the mature CPFhW to approximately 20 μg/g wet weight. This was the highest CPFhW expression level obtained among all the DNA constructs presented in this study.

In eukaryotic organisms, ubiquitin is cleaved out from the fused protein in vivo [62]; hence the antigen administered in the plant material that was transformed with the U::mCPFhW construct was the mature CPFhW alone. In the experiments where CPFhW was used for immunisation, we observed a 50% protection. It is a lower level of protection in comparison with that obtained in experiments where the mature CPFhW fused to HBcAg was used. These results support the use of HBcAg as a carrier for foreign antigens to potentiate protection in the vaccinated animals.

In Experiment III, we observed that the oral vaccination of Sprague-Dawley rats with the lyophilised transgenic lettuce expressing the mCPFhW::G::C or the U::mCPFhW led primarily to a Th2 antibody response against the metacercarial challenge. These enterally immunised rats showed clear IgG1 and IgM responses to CPFhW for 4 consecutive weeks after the challenge, with an increase in the level of IgG1 specific to CPFhW. Clear IgG1, IgM and IgA antibody responses against the ES antigens were visible in all the challenged rats, but the IgM and IgA antibody levels were higher in the vaccinated animals. Also in our previous study [63], we observed significant IgM and IgA antibody responses in immunised rats. Tliba et al. [64] have reported the persistence of a high level of IgM on the teguments of *F*. *hepatica* isolated from rat livers between weeks 1 and 8 post-infection. They speculated that the IgM deposition on the fluke’s tegument might inhibit eosinophil access to the parasite, which would enable it to avoid the antibody-dependent cell-mediated cytotoxicity. Tliba et al. [64] had also observed the presence of IgA and IgE antibodies on the flukes and in the liver tissue surrounding parasites, albeit to a smaller extent. Van Milligen et al. [39,65] have reported that the protective immune responses in immunised rats kill juvenile flukes within the gut wall and peritoneal cavity, and a much smaller percentage of fluke survive gut wall penetration and migration through the peritoneal cavities of immune rats when compared to naïve rats. It has also been shown that in resistant rats, newly excysted juveniles entering the peritoneal cavity are coated with antibodies as well as eosinophils, neutrophils, macrophages and mast cells [66]. This suggests that resistance to flukes in rats may involve both an effective antibody response and lymphoid cells attacking juvenile flukes, with eosinophils and IgG1 correlating with protection at the gut wall [64,66].

We investigated the effectiveness of oral vaccination with plant-produced *F*. *hepatica* CPFhW in rats. Rats are often used as a model to study immunity in cattle due to the fact that the course of infection is similar in both of these animals. In rats, as well as in cattle, infection with *F*. *hepatica* mc causes partial protection to a challenge infection [67–69]. Therefore, the results reported in this study on the oral vaccination with plant-produced *F*. *hepatica* CPFhW are the basis for the development of similar vaccines against *F*. *hepatica* infection in large ruminants. However, some important issue to consider are how the differences in the ruminant gut might affect the vaccine and whether the plant-bioencapsulated antigen would still be viable once the plant material reaches the intestine. Although there are numerous studies reporting immunogenicity of orally delivered plant-made vaccines in humans and small animal models, only a few have successfully demonstrated their efficacy in ruminants [70–74]. The concept of transgenic plant-based vaccines has been successfully employed by Pelosi et al. in their study on antigen-specific antibody responses against a model antigen (the B subunit of the heat labile toxin of enterotoxigenic *E*. *coli*) in sheep following oral immunisation with plant-made and delivered vaccines. The delivery resulted in antigen-specific immune responses in mucosal secretions of the abomasum, small intestine and mesenteric lymph nodes. These findings suggest that the orally administered plant-bioencapsulated antigens are still viable after the passage through the rumen and elicit mucosal and systemic immune responses in sheep [70].

It has been suggested that the loss of production in cattle is significant once the *Fasciola* infection levels establish above 30–40 flukes [75,76]. In sheep, production losses are observed with fluke burdens ranging from 30 to 54 flukes [75,77]. Taking these data into account, it appears that a fluke vaccine would need to reduce the fluke burden below these thresholds to ensure sustainable production benefits. This indicates that the vaccine efficacy required to reduce fluke burdens in sheep and cattle below the threshold varies from approximately 50% to 80% [29].

It has been suggested that due to the emergence of drug-resistant parasites [19–23], combined with the growing consumer concern over chemical residues in food and their passage into the environment, it may be commercially feasible to introduce a fluke vaccine with suboptimal but reasonable efficacy (50%). Such a vaccine would provide economic benefits for dairy producers because it would not compromise fluke control during lactation, and it would leave no residues in the milk [29]. In light of these findings, oral vaccination against *F*. *hepatica* infection with plant-produced antigens is a viable approach due to its convenience and high efficacy without adjuvants.
